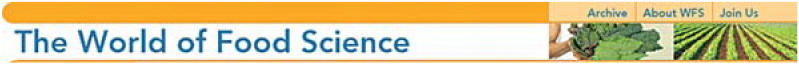# EHPnet: The World of Food Science

**Published:** 2006-06

**Authors:** Erin E. Dooley

The International Union of Food Science and Technology (IUFoST) is a nonprofit network of national food science organizations and is the only global food science and technology group, while the Institute of Food Technologists (IFT) is a U.S. scientific society for food scientists, technologists, and related professionals in industry, academia, and government. These two groups have come together to produce an online magazine, *The World of Food Science*, located at **http://www.worldfoodscience.org/**. With *The World of Food Science*, the IUFoST and the IFT work together to promote the scientific and technical aspects of international food production, distribution, preparation, and marketing, as well as provide current information to all food science professionals and others involved in the industry.

The homepage for *The World of Food Science* provides in-depth Focus and Feature articles collected from a variety of sources, including IUFoST correspondents and food scientists from around the world. Recent articles have covered modern biotechnology in food production, obesity, and how food packaging has evolved to ensure the safety of the food supply. Select articles are available in French and/or Spanish as well as English. Past articles are available by clicking the Archive link at the top of the home-page. The site also includes two Spanish-language sections: the full text of a report titled *Biotecnología y Alimentos* [“Biotechnology and Food”] and IFT Scientific Status Summaries that have been translated from English to Spanish.

At the bottom of the homepage are links to documents and expert reports published by the IFT. Currently featured are annual buyer’s guides for nutraceuticals (foods or food components that provide a medical or health benefit) and food industry services. Visitors will also find links to the expert panel reports *Functional Foods: Opportunities and Challenges* (“functional foods” are formulated with ingredients believed to impart health benefits) and *Emerging Microbiological Food Safety Issues: Implications for Control in the 21st Century*. Clicking on the links for the expert reports takes visitors to webpages devoted solely to those respective topics. Available resources include the full text of each expert panel review, one-page topic summaries, related news releases, and lists of frequently asked questions.

The homepage also includes a frequently updated collection of news coverage. Clicking on any headline takes the visitor to the Daily News page. A searchable library of past news articles is archived by month. Visitors can also choose from a menu of other news options: national food technology association news, regional reports on food issues, papers directed toward students in the field, international regulatory updates, and policy papers. The Events section has information on upcoming meetings related to food science and technology.

## Figures and Tables

**Figure:**